# The role of immune- and lipid metabolism-related genes in macrophage polarization and prognosis of glioblastoma

**DOI:** 10.3389/fonc.2025.1660754

**Published:** 2025-10-14

**Authors:** Yue Zhang, Xin Xu, Shuo Li, Chenlong Liao, Xiaosheng Yang, Wenchuan Zhang

**Affiliations:** Department of Neurosurgery, Shanghai Ninth People’s Hospital, School of Medicine, Shanghai Jiao Tong University, Shanghai, China

**Keywords:** glioblastoma, macrophage polarization, lipid metabolism, prognostic genes, immune response

## Abstract

**Objective:**

To investigate the roles of immune- and lipid metabolism-related genes in macrophage polarization and their prognostic and therapeutic implications in glioblastoma (GBM).

**Methods:**

A total of 655 GBM samples from The Cancer Genome Atlas (TCGA) were stratified into immune and non-immune groups based on immune scores. Differentially expressed genes (DEGs) were identified, and their intersection with 859 lipid metabolism–related genes yielded 26 candidates. A 10-gene prognostic signature was constructed using univariate and least absolute shrinkage and selection operator (LASSO) Cox regression analyses and validated in both internal (TCGA) and independent (Chinese Glioma Genome Atlas, CGGA) cohorts. Functional enrichment, single-cell transcriptomic analysis, experimental validation, and drug sensitivity profiling were performed to assess the biological and therapeutic relevance of the identified genes.

**Results:**

Ten immune- and lipid metabolism–related genes were significantly associated with GBM prognosis. Key genes such as *LGALS1*, *PLA2G5*, and *FABP5* were upregulated in high-risk patients and enriched in M2-like tumor-associated macrophages. Enrichment analyses indicated their involvement in immune regulation and lipid metabolic pathways. Their elevated expression in GBM tissues was confirmed by qRT-PCR and Western blot. Drug sensitivity analysis demonstrated a correlation between *LGALS1* expression and the response to agents such as zoledronate and staurosporine.

**Conclusions:**

Immune- and lipid metabolism–related genes contribute to macrophage polarization and are closely linked to GBM prognosis. The identified gene signature provides prognostic value and potential therapeutic targets for immunometabolic modulation in GBM.

## 1 Introduction

Glioblastoma (GBM) is an aggressive primary brain tumor marked by dismal outcomes despite multimodal therapy. The 5-year survival rate is only around 5%, reflecting the failure of current treatments to control this malignancy (1). A major challenge in GBM management is the extreme heterogeneity of the tumor at both the molecular and cellular levels (2). GBMs comprise multiple subtypes and evolving niches within a single tumor, leading to varied therapeutic responses and fostering treatment resistance. This heterogeneity extends to the tumor microenvironment (TME), which in GBM is highly immunosuppressive and poses a barrier to immunotherapy (3, 4). Immunotherapeutic strategies that succeed in other cancers have shown limited benefit in GBM, in large part due to the unique immune milieu of the brain tumor and its capacity to evade anti-tumor immune responses. Thus, there is an urgent need for new biomarkers and models that capture the complex biology of GBM and improve prognostic and therapeutic stratification.

One key element of GBM’s TME is the abundance of tumor-associated macrophages (TAMs), which can constitute 30–50% of the cellular content of the tumor (5). TAMs in GBM originate from both brain-resident microglia and infiltrating monocytes, and together they dominate the immune landscape and drive immunosuppression. Notably, increased TAM infiltration correlates with tumor growth, recurrence, and worse patient survival (6). TAMs exhibit remarkable plasticity, generally polarized between two ends of a spectrum: the pro-inflammatory “classically activated” M1 phenotype and the anti-inflammatory “alternatively activated” M2 phenotype (7). M1-polarized macrophages can phagocytose tumor cells and stimulate immune responses, exerting anti-tumor effects, whereas M2-polarized TAMs promote tumor cell proliferation, angiogenesis, and tissue remodeling, thereby facilitating GBM progression (8). In GBM, TAMs tend to skew towards the M2-like state, and a high M2/M1 ratio has been associated with immunotherapy resistance and poor prognosis. M2-like TAMs secrete immunosuppressive cytokines (e.g. IL-10, TGF-β) and growth factors (e.g. VEGF) that blunt cytotoxic T cell activity and support tumor growth, effectively creating a protective niche for the tumor (9). This TAM-driven immunosuppressive microenvironment is a major hurdle for immune-based therapies and contributes to the failure of GBM to respond to checkpoint inhibitors and other immunotherapies.

Beyond well-characterized genetic alterations, GBM tumor biology is profoundly influenced by metabolic reprogramming and its crosstalk with immune elements of the TME. Emerging evidence indicates that metabolic disturbances in GBM – including aberrant lipid metabolism – can shape the immune contexture of the tumor (10, 11). Tumor cells in GBM upregulate pathways for glycolysis, glutamine utilization, and lipid synthesis/oxidation to survive in hypoxic, nutrient-deprived conditions. These metabolic changes not only fuel tumor growth but also impair immune cell function, driving T cell exhaustion and biasing myeloid cells toward immunosuppressive phenotypes. In particular, the polarization of macrophages is closely tied to their metabolic state: M1 macrophages rely on aerobic glycolysis, whereas M2 macrophages depend on oxidative metabolism such as fatty acid oxidation (12, 13). The lipid-rich environment of GBM – partly a result of tumor cell necrosis and active lipid biosynthesis – can thus preferentially support an M2-like TAM phenotype. Indeed, tumor-derived lipids and metabolic signals can hijack macrophage programming; for example, high uptake of fatty acids via the scavenger receptor CD36 drives macrophages toward an M2 state (14). Consistent with this, *in situ* studies of human GBM have identified “foam cell” TAMs engorged with lipid droplets in perinecrotic regions, where they enhance tumor-promoting conditions (15). These lipid-laden macrophages secrete angiogenic factors like VEGF and HGF under hypoxia and further dampen immunity by recruiting additional M2 macrophages and inhibiting T cell responses. The presence of such lipid-loaded TAMs is associated with an immunosuppressive, pro-tumoral niche in GBM and highlights a direct link between disordered lipid metabolism and immune dysfunction in the tumor. Furthermore, glioma-associated myeloid cells can supply metabolic resources to tumor cells; for instance, macrophages and microglia in GBM can recycle myelin debris and release lipids that are taken up by tumor cells to support their growth and a mesenchymal, aggressive phenotype (16, 17). Altogether, these findings underscore that the interplay between lipid metabolism and the immune microenvironment is a crucial but underexplored facet of GBM pathogenesis.

Given the contributions of both immune suppression and metabolic reprogramming to GBM malignancy, we hypothesized that integrating immune-related and lipid metabolism–related factors could yield novel prognostic insights. In this study, we aimed to develop a prognostic model based on genes involved in immune regulation and lipid metabolism, to better capture the combined effect of tumor immunity and metabolism on patient outcomes. The rationale is that such a gene signature might reflect the degree of “immunometabolic” dysregulation in each tumor – for example, the extent of M2 macrophage skewing or lipid-fueled tumor aggressiveness – and thereby stratify patients by survival risk. We constructed and validated an immune-lipid gene signature in GBM cohorts, and explored its association with overall survival. We further examined how the risk signature correlates with the tumor immune microenvironment, particularly the polarization state of TAMs (M1 vs M2), as well as with potential therapeutic vulnerabilities such as sensitivity to drugs. Our objective was to provide a more comprehensive prognostic tool that not only predicts outcomes but also offers biological insights into macrophage polarization and metabolic targets in GBM, ultimately paving the way for improved therapeutic strategies.

## 2 Materials and methods

### 2.1 Data sources and sample acquisition

Gene expression profiles and clinical annotations of diffuse gliomas were retrieved from The Cancer Genome Atlas (TCGA) and the Chinese Glioma Genome Atlas (CGGA). A total of 1,528 glioma samples, encompassing both lower-grade gliomas (WHO grade II–III) and GBM (WHO grade IV), were included. Samples with missing clinical data were excluded. The TCGA cohort (n = 655) was randomly divided into a training set (~60%) and an internal validation set (~40%) using stratified sampling based on tumor grade. The CGGA dataset (n = 873) served as an external validation cohort.

Single-cell transcriptomic data were obtained from the GEO dataset GSE84465, comprising 3,589 cells isolated from four primary GBM specimens. These data were used to analyze TME composition and cell-type–specific gene expression patterns.

A comprehensive list of 859 lipid metabolism–related genes was compiled from the Molecular Signatures Database (MSigDB), including Reactome, Hallmark, and KEGG lipid-related gene sets. These genes were intersected with immune-related differentially expressed genes (DEGs) to obtain a final set of 26 candidates used for prognostic model construction.

### 2.2 Differential gene expression analysis

Based on immune score distribution, glioma samples were classified into immune-infiltrated and non-infiltrated groups. Differential expression analysis between these groups was performed using the “limma” package in R. Genes with an absolute log_2_ fold change > 1 and a false discovery rate (FDR) < 0.05 were considered significantly differentially expressed.

### 2.3 Construction of the prognostic gene signature

**Figure 1:**
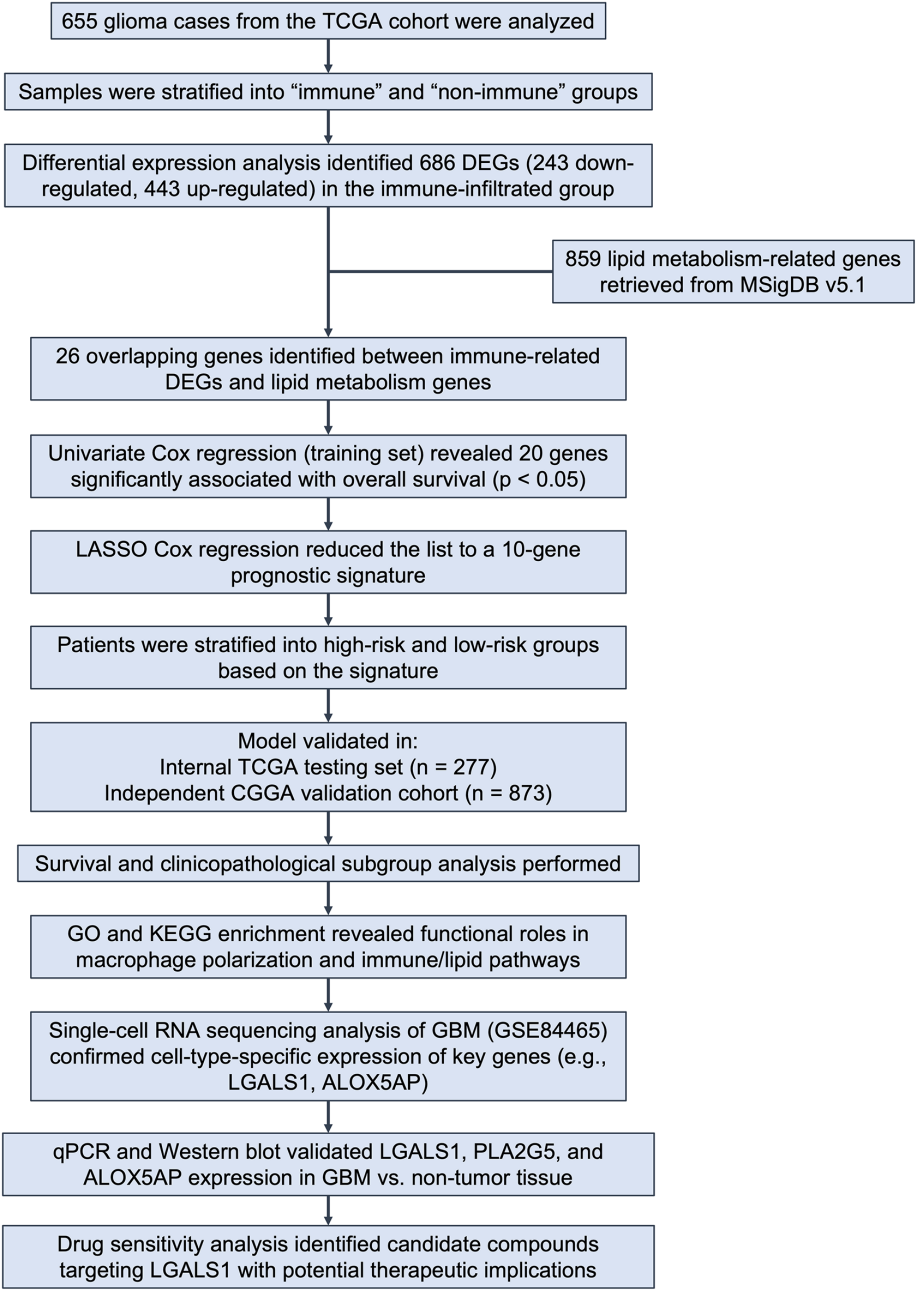
Overview of study design and analysis workflow. **Figure 1** shows the analytical workflow of this study. Glioma samples from the TCGA cohort (n = 655) were classified into immune and non-immune groups based on immune scores. DEGs were identified, and 26 genes overlapping with 859 lipid metabolism–related genes from MSigDB were selected. Univariate and LASSO Cox regression analyses yielded a 10-gene prognostic signature, which stratified patients into high- and low-risk groups. The model was validated in both the TCGA testing set and an external CGGA cohort. Further analyses included survival and subgroup assessment, functional enrichment, single-cell transcriptomic profiling, experimental validation, and drug sensitivity analysis.

Univariate Cox proportional hazards regression was first applied to the 26 immune- and lipid-related DEGs to screen for genes significantly associated with overall survival (p < 0.05). These candidate genes were then subjected to least absolute shrinkage and selection operator (LASSO) Cox regression using the “glmnet” R package to avoid overfitting. A prognostic signature comprising 10 genes was constructed based on the optimal penalty parameter (λ). Risk scores were calculated for each patient as a weighted sum of normalized gene expression values multiplied by their respective LASSO coefficients. Patients were stratified into high- and low-risk groups using the median risk score as the cutoff. **Figure 1** presents the workflow of this study.

### 2.4 Evaluation and validation of the prognostic model

Kaplan–Meier survival analysis and the log-rank test were used to compare overall survival between the high- and low-risk groups. Time-dependent receiver operating characteristic (ROC) curves and corresponding area under the curve (AUC) values were generated to evaluate the predictive accuracy of the model at 1-, 3-, and 5-year intervals. The performance of the risk model was validated across the TCGA internal validation set and the CGGA external cohort. Univariate and multivariate Cox regression analyses were conducted to assess whether the risk score was an independent prognostic factor.

### 2.5 Functional enrichment analysis

Gene Ontology (GO) and Kyoto Encyclopedia of Genes and Genomes (KEGG) enrichment analyses were performed on the genes included in the prognostic signature using the “clusterProfiler” package in R. GO terms and KEGG pathways with an adjusted p-value < 0.05 (FDR corrected) were considered significantly enriched. Biological processes related to immune regulation, macrophage activation, and lipid metabolism were particularly emphasized.

### 2.6 Single-cell RNA-sequencing analysis

Quality control, normalization, dimensionality reduction, and clustering of the GSE84465 dataset were conducted using the Seurat package (v4.0.4) in R (v4.2.2). Cells with fewer than 200 detected genes, more than 2,500 detected genes, or >10% mitochondrial gene content were excluded to remove low-quality cells. Gene expression values were normalized using the “LogNormalize” method with a scale factor of 10,000, and the top 2000 variable genes were identified for downstream analyses.

Principal component analysis (PCA) was performed, and the top 30 PCs were used to construct a shared nearest neighbor (SNN) graph. Unsupervised clustering was conducted via the Louvain algorithm, and clusters were visualized using both t-distributed stochastic neighbor embedding (t-SNE) and Uniform Manifold Approximation and Projection (UMAP).

Marker genes for each cluster were identified using the “FindMarkers” function, and cluster annotation combined manual curation based on canonical markers (e.g., CD3D and CD3E for T cells, CD68 and CSF1R for macrophages, GFAP for astrocytes) with reference-based validation using the CellMarker database. To further confirm annotation accuracy, we cross-referenced with previously published GBM single-cell atlases and visualized marker expression patterns using violin and feature plots.

The expression of selected prognostic genes (e.g., *ALOX5AP, LGALS1, PLA2G5*) was examined to determine cell-type specificity, particularly within macrophage subpopulations. Differential expression and pathway enrichment analyses were also conducted to support biological interpretation.

### 2.7 Immune infiltration analysis

The CIBERSORT algorithm was applied to bulk RNA-sequencing data from the TCGA cohort using the LM22 reference signature to estimate the relative abundance of 22 immune cell types. Only samples with a CIBERSORT p-value < 0.05 were included in downstream analyses. The proportions of M1- and M2-like macrophages were compared between high- and low-risk groups using the Wilcoxon rank-sum test. The M1/M2 ratio was also calculated and its correlation with risk score was assessed using Spearman’s rank correlation.

### 2.8 Experimental validation

To validate the expression levels of selected risk genes, GBM and non-tumor brain tissue samples were collected during neurosurgical procedures (e.g., epilepsy or trauma surgery). All patients provided informed consent, and the study was approved by the institutional ethics committee.

Quantitative reverse transcription polymerase chain reaction (qRT-PCR): Total RNA was extracted using TRIzol reagent (Invitrogen, USA) and quantified using a NanoDrop spectrophotometer. Complementary DNA (cDNA) was synthesized using a commercial reverse transcription kit (manufacturer to be specified). Quantitative real-time polymerase chain reaction (PCR) was performed on 15 pairs of GBM and adjacent non-tumor brain tissue samples using an ABI 7500 system using SYBR Green Master Mix. β-actin served as the internal control. Reactions were run in triplicate, and gene expression levels were calculated using the 2^–ΔΔCt method. The primer sequences employed for PCR amplification are detailed in the **Supplementary Material** accompanying this study.

Western Blot: Total protein was extracted from tissue samples using RIPA buffer supplemented with protease inhibitors. Equal amounts of protein were separated by SDS-PAGE and transferred to PVDF membranes (Millipore). Membranes were blocked and incubated overnight at 4 °C with primary antibodies (e.g., anti-Nmb, anti-NmbR; manufacturer and dilution to be specified), followed by HRP-conjugated secondary antibodies. Protein bands were visualized using enhanced chemiluminescence (ECL) and quantified using ImageJ software. β-actin was used as a loading control.

The reverse transcription kit (Name, Manufacturer, Catalog number) and antibodies used for Western blot (target, manufacturer, catalog number, dilution) are provided in **Supplementary Table 4** and **5**.

### 2.9 Drug sensitivity analysis

Drug response data were obtained from the Genomics of Drug Sensitivity in Cancer (GDSC) database. The “pRRophetic” package was used to estimate the half-maximal inhibitory concentration (IC50) values of chemotherapeutic and targeted agents based on gene expression profiles. Pearson correlation was used to evaluate the association between gene expression (e.g., *LGALS1*) and predicted drug sensitivity. Differences in IC50 values between risk groups were assessed using Student’s t-test.

### 2.10 Statistical analysis

All statistical analyses were performed using R software (v4.2.2). The Wilcoxon rank-sum test was applied to compare numerical variables between two groups, such as immune, stromal, and ESTIMATE scores, as well as immune cell proportions. Pearson or Spearman correlation analyses were applied based on data distribution to evaluate associations between gene expression, immune cell infiltration, drug sensitivity, and risk scores.

Survival analyses were conducted using the Kaplan–Meier method and log-rank test. Univariate and multivariate Cox proportional hazards regression models were used to identify independent prognostic factors. Time-dependent ROC curves were constructed using the “survivalROC” package to assess the predictive performance of the risk model.

The sample size of experimental validation was determined based on clinical tissue availability and comparable studies in the field, which is a common approach in glioblastoma research. To further ensure robustness, we expanded the qRT-PCR validation cohort to 15 pairs of GBM and non-tumor tissues in the revised manuscript. Hazard ratios (HRs) with 95% confidence intervals (CIs) were reported for survival analyses, and area under the ROC curve (AUC) values were provided to quantify the predictive performance of the model.

Enrichment analyses were adjusted using the Benjamini–Hochberg method for multiple testing correction. All statistical tests were two-sided, and p-values < 0.05 were considered statistically significant.

## 3 Manuscript formatting

### 3.1 Patient sample classification and baseline characteristics of datasets

To construct and validate the prognostic model, a total of 655 glioma samples from TCGA were included. These samples were randomly divided into a training set (n = 378) and a testing set (n = 277) using a 3:2 ratio. Pathological classification was performed according to the 2021 WHO criteria, in which tumors diagnosed as grade IV gliomas were designated as GBM, while those with grade II–III were categorized as non-GBM gliomas.

The clinical and demographic characteristics of patients in the TCGA training and testing sets are summarized in **Table 1**. No significant differences were observed between the two groups with respect to age distribution, gender, or histological subtype, confirming the comparability of the datasets for model training and internal validation.

**Table 1:** Baseline demographic characteristic of glioma (TCGA).

Variables	Training set (n=378)	Testing set (n=277)	P value
Age, n (%)			0.557
<45	177 (46.8%)	137 (49.5%)	
≥45	201 (53.2%)	140 (50.5%)	
Gender			0.901
Female	158 (41.8%)	118 (42.6%)	
Male	220 (58.2%)	159 (57.4%)	
Race			0.939
Asian	9 (2.38%)	5 (1.81%)	
Black or African American	19 (5.03%)	12 (4.33%)	
White	344 (91.0%)	255 (92.1%)	
Not reported	6 (1.59%)	5 (1.81%)	
Grade			0.252
G2	144 (38.1%)	100 (36.1%)	
G3	141 (37.3%)	120 (43.3%)	
G4	93 (24.6%)	57 (20.6%)	
Type			0.264
Non-GBM	285 (75.4%)	220 (79.4%)	
GBM	93 (24.6%)	57 (20.6%)	
Radiation therapy			0.476
NO	106 (28.0%)	81 (29.2%)	
YES	238 (63.0%)	164 (59.2%)	
Unknown	34 (8.99%)	32 (11.6%)	
Neoadjuvant treatment			0.577
No	377 (99.7%)	275 (99.3%)	
Yes	1 (0.26%)	2 (0.72%)	
New tumor event after initial treatment			0.3
NO	152 (40.2%)	118 (42.6%)	
YES	68 (18.0%)	62 (22.4%)	
Unknown	158 (41.8%)	97 (35.0%)	
Sample type			0.732
Primary Tumor	367 (97.1%)	271 (97.8%)	
Recurrent Tumor	11 (2.91%)	6 (2.17%)	
Primary site			0.035
Brain, NOS	98 (25.9%)	60 (21.7%)	
Frontal Lobe	154 (40.7%)	143 (51.6%)	
Occipital Lobe	7 (1.85%)	1 (0.36%)	
Parietal Lobe	32 (8.47%)	14 (5.05%)	
Posterior Fossa, Cerebellum	1 (0.26%)	1 (0.36%)	
Temporal Lobe	86 (22.8%)	58 (20.9%)	
Laterality			0.492
Left	143 (37.8%)	104 (37.5%)	
Midline	2 (0.53%)	3 (1.08%)	
Right	140 (37.0%)	113 (40.8%)	
Unknown	93 (24.6%)	57 (20.6%)	
First presenting symptom			-
Seizures	136 (36.0%)	109 (39.4%)	
Headaches	54 (14.3%)	49 (17.7%)	
Visual Changes	9 (2.38%)	2 (0.72%)	
Sensory Changes	12 (3.17%)	5 (1.81%)	
Mental Status Changes	20 (5.29%)	19 (6.86%)	
Motor/Movement Changes	23 (6.08%)	14 (5.05%)	
Unknown	124 (32.8%)	79 (28.54%)	
Seizure history			0.347
NO	94 (24.9%)	80 (28.9%)	
YES	169 (44.7%)	129 (46.6%)	
Unknown	115 (30.42%)	68 (24.57%)	
Headache history			0.209
NO	163 (43.1%)	126 (45.5%)	
YES	89 (23.5%)	78 (28.2%)	
Unknown	126 (33.33%)	73 (26.38%)	
Visual changes			0.379
NO	213 (56.3%)	174 (62.8%)	
YES	37 (9.79%)	26 (9.39%)	
Unknown	128 (33.86%)	77 (27.82%)	
Sensory changes			0.092
NO	202 (53.4%)	175 (63.2%)	
YES	44 (11.6%)	24 (8.66%)	
Unknown	132 (34.9%)	78 (28.18%)	
Mental status changes			0.464
NO	189 (50.0%)	149 (53.8%)	
YES	62 (16.4%)	51 (18.4%)	
Unknown	127 (33.59%)	77 (27.82%)	

For external validation, an independent cohort of 655 glioma patients from the CGGA was used. Pathological classification into GBM and non-GBM groups followed the same criteria. The baseline clinical characteristics of the CGGA cohort are presented in **Table 2**.

**Table 2:** Baseline demographic characteristic of glioma (CGGA).

Variables	GBM training set (n=93)	GBM testing set (n=57)	P value	Non-GBM training set (n=301)	Non-GBM testing set (n=204)	P value
Age, n (%)			1			0.779
<45	11 (11.83%)	6 (10.53%)		175 (58.14%)	122 (59.80%)	
≥45	82 (88.17%)	51 (89.47%)		126 (41.86%)	82 (40.20%)	
Gender, n (%)			0.858			0.743
Female	32 (34.41%)	18 (31.58%)		137 (45.51%)	89 (43.63%)	
Male	61 (65.59%)	39 (68.42%)		164 (54.49%)	115 (56.37%)	
Race, n (%)			0.848			0.052
Asian	3 (3.23%)	2 (3.51%)		3 (1.00%)	6 (2.94%)	
Black or African American	5 (5.38%)	5 (8.77%)		15 (4.98%)	6 (2.94%)	
White	84 (90.32%)	50 (87.72%)		274 (91.03%)	191 (93.63%)	
Not reported	1 (1.08%)	0 (0.00%)		9 (2.99%)	1 (0.49%)	
Grade, n (%)			-			1
G2	0 (0.00%)	0 (0.00%)		145 (48.17%)	99 (48.53%)	
G3	0 (0.00%)	0 (0.00%)		156 (51.83%)	105 (51.47%)	
G4	93 (100.00%)	57 (100.00%)		0 (0.00%)	0 (0.00%)	
Radiation therapy, n (%)			1			0.372
NO	13 (13.98%)	8 (14.04%)		94 (31.23%)	72 (35.29%)	
YES	80 (86.02%)	49 (85.96%)		163 (54.15%)	110 (53.92%)	
Unknown	0 (0.00%)	0 (0.00%)		44 (14.62%)	22 (10.78%)	
Neoadjuvant treatment, n (%)			-			1
No	93 (100.00%)	57 (100.00%)		299 (99.34%)	203 (99.51%)	
Yes	0 (0.00%)	0 (0.00%)		2 (0.66%)	1 (0.49%)	
New tumor event after initial treatment, n (%)			-			0.854
NO	0 (0.00%)	0 (0.00%)		164 (54.49%)	106 (51.96%)	
YES	0 (0.00%)	0 (0.00%)		76 (25.25%)	54 (26.47%)	
Unknown	93 (100.00%)	57 (100.00%)		61 (20.27%)	44 (21.57%)	
Sample type, n (%)			1			1
Primary Tumor	87 (93.55%)	54 (94.74%)		296 (98.34%)	201 (98.53%)	
Recurrent Tumor	6 (6.45%)	3 (5.26%)		5 (1.66%)	3 (1.47%)	
Primary site, n (%)			-			0.96
Frontal Lobe	0 (0.00%)	0 (0.00%)		181 (60.13%)	116 (56.86%)	
Occipital Lobe	0 (0.00%)	0 (0.00%)		5 (1.66%)	3 (1.47%)	
Parietal Lobe	0 (0.00%)	0 (0.00%)		25 (8.31%)	21 (10.29%)	
Posterior Fossa, Cerebellum	0 (0.00%)	0 (0.00%)		1 (0.33%)	1 (0.49%)	
Temporal Lobe	0 (0.00%)	0 (0.00%)		84 (27.91%)	60 (29.41%)	
Brain, NOS	93 (100.00%)	57 (100.00%)		5 (1.66%)	3 (1.47%)	
Laterality, n (%)			-			0.487
Left	0 (0.00%)	0 (0.00%)		144 (47.84%)	103 (50.49%)	
Midline	0 (0.00%)	0 (0.00%)		2 (0.66%)	3 (1.47%)	
Right	0 (0.00%)	0 (0.00%)		155 (51.50%)	98 (48.04%)	
Unknown	93 (100.00%)	57 (100.00%)		0 (0.00%)	0 (0.00%)	
First presenting symptom, n (%)			-			0.78
Seizures	0 (0.00%)	0 (0.00%)		145 (48.17%)	100 (49.02%)	
Headaches	0 (0.00%)	0 (0.00%)		61 (20.27%)	42 (20.59%)	
Visual Changes	0 (0.00%)	0 (0.00%)		5 (1.66%)	6 (2.94%)	
Sensory Changes	0 (0.00%)	0 (0.00%)		9 (2.99%)	8 (3.92%)	
Mental Status Changes	0 (0.00%)	0 (0.00%)		26 (8.64%)	13 (6.37%)	
Motor/Movement Changes	0 (0.00%)	0 (0.00%)		25 (8.31%)	12 (5.88%)	
Unknown	93 (100.00%)	57 (100.00%)		30 (9.97%)	23 (11.27%)	
Seizure history, n (%)			-			0.875
NO	0 (0.00%)	0 (0.00%)		101 (33.55%)	73 (35.78%)	
YES	0 (0.00%)	0 (0.00%)		180 (59.80%)	118 (57.84%)	
Unknown	93 (100.00%)	57 (100.00%)		20 (6.64%)	13 (6.37%)	
Headache history, n (%)			-			0.879
NO	0 (0.00%)	0 (0.00%)		174 (57.81%)	115 (56.37%)	
YES	0 (0.00%)	0 (0.00%)		97 (32.23%)	70 (34.31%)	
Unknown	93 (100.00%)	57 (100.00%)		30 (9.97%)	19 (9.31%)	
Visual changes, n (%)			-			0.765
NO	0 (0.00%)	0 (0.00%)		230 (76.41%)	157 (76.96%)	
YES	0 (0.00%)	0 (0.00%)		36 (11.96%)	27 (13.24%)	
Unknown	93 (100.00%)	57 (100.00%)		35 (11.63%)	20 (9.80%)	
Sensory changes, n (%)			-			0.483
NO	0 (0.00%)	0 (0.00%)		219 (72.76%)	158 (77.45%)	
YES	0 (0.00%)	0 (0.00%)		43 (14.29%)	25 (12.25%)	
Unknown	93 (100.00%)	57 (100.00%)		39 (12.96%)	21 (10.29%)	
Mental status changes, n (%)			-			0.54
NO	0 (0.00%)	0 (0.00%)		196 (65.12%)	142 (69.61%)	
YES	0 (0.00%)	0 (0.00%)		70 (23.26%)	43 (21.08%)	
Unknown	93 (100.00%)	57 (100.00%)		35 (11.63%)	19 (9.31%)	

This multi-cohort design allowed for robust evaluation of the prognostic performance and generalizability of the model across distinct populations.

### 3.2 Association between ESTIMATE scores and clinicopathological features

**Figure 2:**
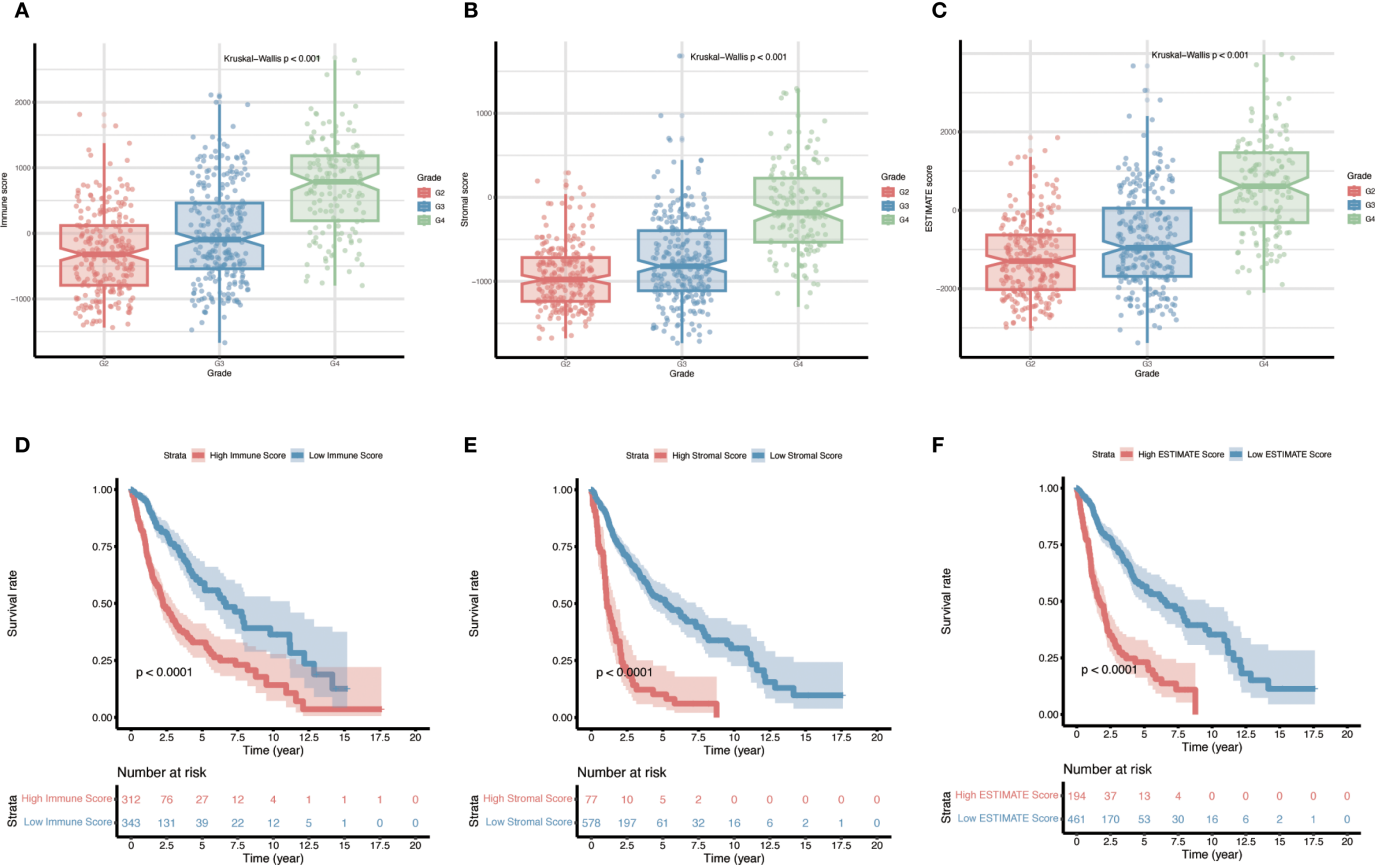
Relationship between immune, stromal, and ESTIMATE scores and prognosis in gliomas. **(A–C)** Distributions of stromal scores, immune scores, and composite ESTIMATE scores across different glioma pathological grades (p < 0.001 for all grade comparisons). **(D–F)** Kaplan–Meier overall survival curves for patients stratified into high-score vs. low-score groups (for Stromal, Immune, and ESTIMATE scores respectively), with p-values determined by log-rank tests.

A total of 655 cases from the TCGA glioma cohort were analyzed, and for each sample the stromal, immune, and ESTIMATE scores were calculated (ranges: –1735.34 to 1682.27, –1669.12 to 2679.67, and –3381.83 to 3974.18, respectively). All three scores were significantly correlated with tumor grade: WHO grade IV tumors (GBM) exhibited higher immune, stromal, and ESTIMATE scores than lower-grade (WHO II–III) tumors (**Figures 2A–C**, p, < 0.001). Furthermore, patients with higher stromal, immune, or ESTIMATE scores had significantly worse overall survival compared to those with lower scores (**Figures 2D–F**).

### 3.3 Identification of DEGs

**Figure 3:**
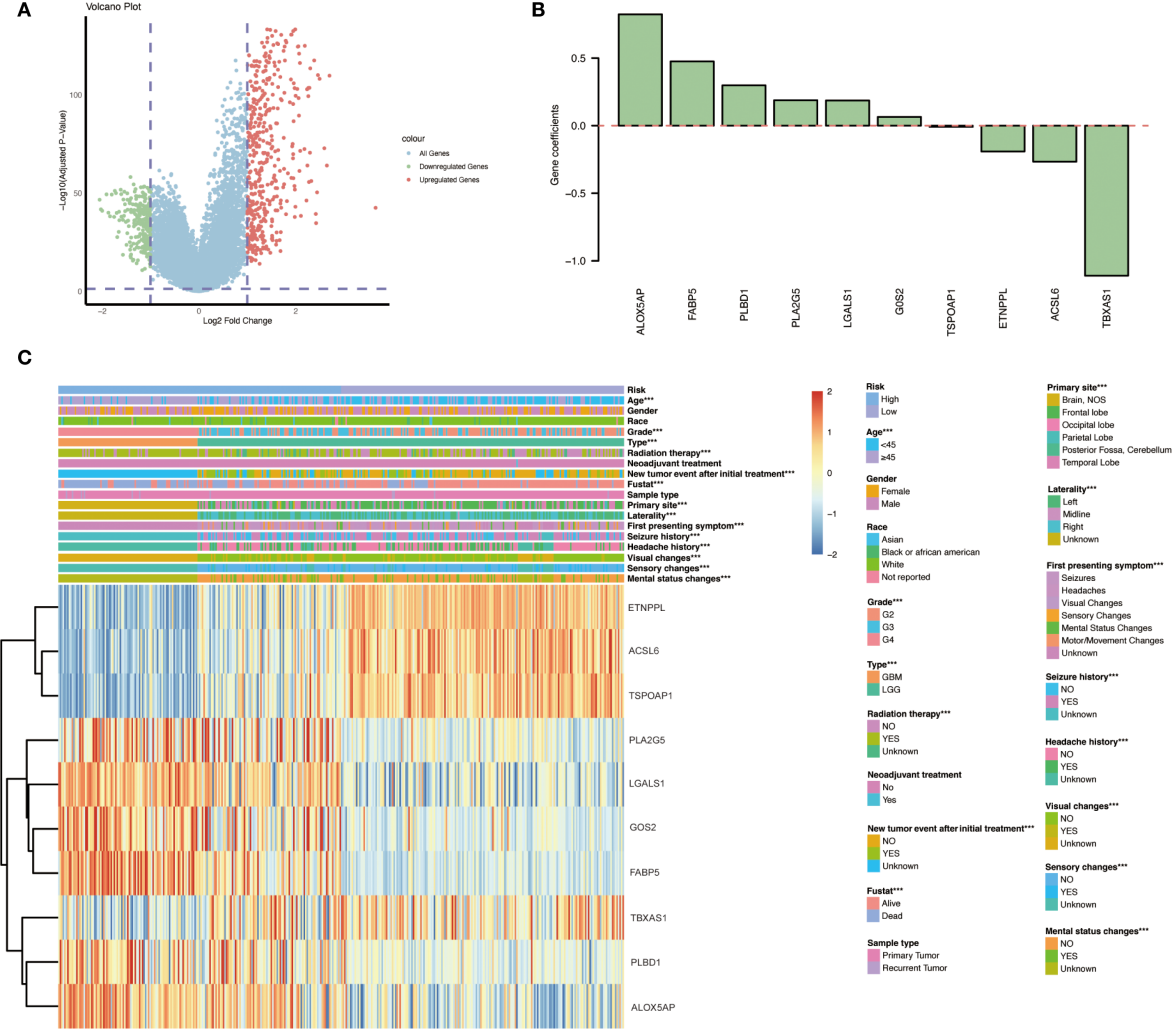
Differential gene expression and risk gene profiles between immune-infiltrated and non-infiltrated gliomas. **(A)** Volcano plot illustrating the distribution of DEGs between the immune group and non-immune group, highlighting 243 down-regulated and 443 up-regulated genes in the immune-infiltrated group. **(B)** Gene coefficients. **(C)** Heatmap presenting the expression levels of the ten identified immune- and lipid metabolism–related risk genes across various glioma patient subgroups with different clinicopathological characteristics and outcomes. Significant expression differences between subgroups are indicated: *p < 0.05; **p < 0.01; ***p < 0.001.

Using the immune score to stratify cases, the cohort was divided into an “immune” group (positive immune score) and a “non-immune” group (negative immune score). Differential expression analysis between these groups identified a total of 686 DEGs, comprising 243 down-regulated genes and 443 up-regulated genes in the immune-infiltrated group (**Figure 3A**). These genes represent the immune-related transcriptomic differences between immune-high and immune-low gliomas. The overall distribution of the DEGs is visualized in **Figure 3A** (volcano plot), highlighting the magnitude of expression changes between the two groups.

### 3.4 Overlap with lipid metabolism-related genes

To narrow down the candidates, we intersected the immune-related DEGs with genes involved in lipid metabolism. A set of 859 lipid metabolism-related genes was compiled from the MSigDB v5.1 (including Reactome pathways for lipid and phospholipid metabolism, the Hallmark fatty acid metabolism gene set, and KEGG glycerophospholipid metabolism pathways). The overlap between these 859 genes and the 686 immune-related DEGs yielded 26 genes that are related to both immune response and lipid metabolism. These 26 overlapping genes were taken forward as candidate genes potentially involved in immune regulation and lipid metabolic processes in glioma.

### 3.5 Development and validation of the prognostic model

**Figure 4:**
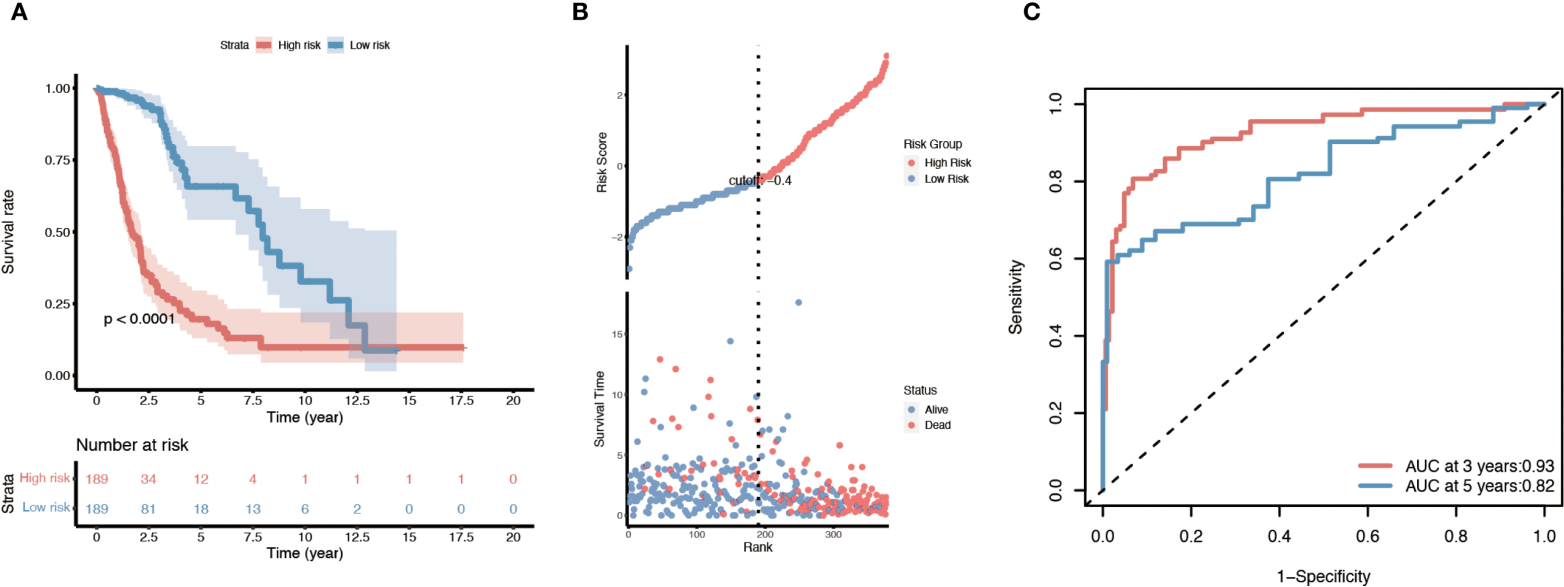
Survival analysis of the ten-gene immune/lipid metabolism signature in the TCGA glioma cohort. **(A)** Kaplan–Meier overall survival curve comparing patients in the high-risk vs. low-risk groups defined by the ten-gene risk score. **(B)** Distribution of risk scores (based on the ten-gene signature) along with each patient’s survival status and time; patients with lower risk scores tend to have longer survival. **(C)** Time-dependent ROC curves evaluating the prognostic performance of the risk model at 3 and 5 years, with the AUC values for 3-year and 5-year survival indicated. Log-rank p-values are shown for the survival differences between risk groups.

We next constructed a prognostic model based on the overlapping immune- and lipid metabolism-related genes. First, univariate Cox regression in the training set identified 20 candidate genes (out of the 26 overlap genes) significantly associated with overall survival (*p* < 0.05). These were further narrowed down via LASSO Cox regression, resulting in a signature of 10 key risk genes. Using the expression of these ten genes, a risk score was calculated for each patient, and the cohort was split into high-risk and low-risk groups (typically using the median risk score as the cutoff). Kaplan–Meier survival analysis revealed that patients in the high-risk group had a markedly poorer prognosis than those in the low-risk group (**Figure 4A**). Consistently, visualization of the risk score distribution, survival time, and survival status demonstrated that lower risk scores tended to be associated with longer survival durations (**Figure 4B**). Time-dependent ROC analysis further showed that the prognostic model achieved favorable accuracy, with appreciable 3-year and 5-year AUC values for overall survival prediction (**Figure 4C**).

The prognostic value of this 10-gene risk signature was validated in both internal and external datasets (**Supplementary Figure 1**). In the TCGA internal testing set, the same trend was observed: high-risk patients had significantly shorter survival compared to low-risk patients, mirroring the training set results (**Supplementary Figure 1A–C**). Similarly, when applied to the independent CGGA cohort, the risk score stratification successfully distinguished outcomes, with the high-risk group demonstrating worse overall survival than the low-risk group (consistent with **Figure 4A**) (**Supplementary Figure 1D–F**). These concordant results across different datasets confirm the robustness and generalizability of the prognostic model.

### 3.6 Expression analysis and identification of independent risk factors

We examined the expression patterns and clinical associations of the ten risk signature genes in glioma patients. Notably, distinct subsets of these genes were associated with opposite prognostic implications. Six genes — *PLA2G5*, *LGALS1*, *GOS2*, *FABP5*, *PLBD1*, and *ALOX5AP* — were identified as “risk genes,” meaning that higher expression levels of these genes correlated with poorer patient outcomes. In contrast, the remaining four genes — *ETNPPL*, *ACSL6*, *TSPOAP1*, and *TBXAS1* — behaved as “protective genes,” where elevated expression was associated with improved survival (**Figure 3C**). Furthermore, three of the protective genes (ETNPPL, ACSL6, and TSPOAP1) were found to be significantly down-regulated in the tumors of WHO grade IV as compared to lower grades, suggesting that loss of these genes’ expression may be linked to the development of more aggressive gliomas (GBM).

**Figure 5:**
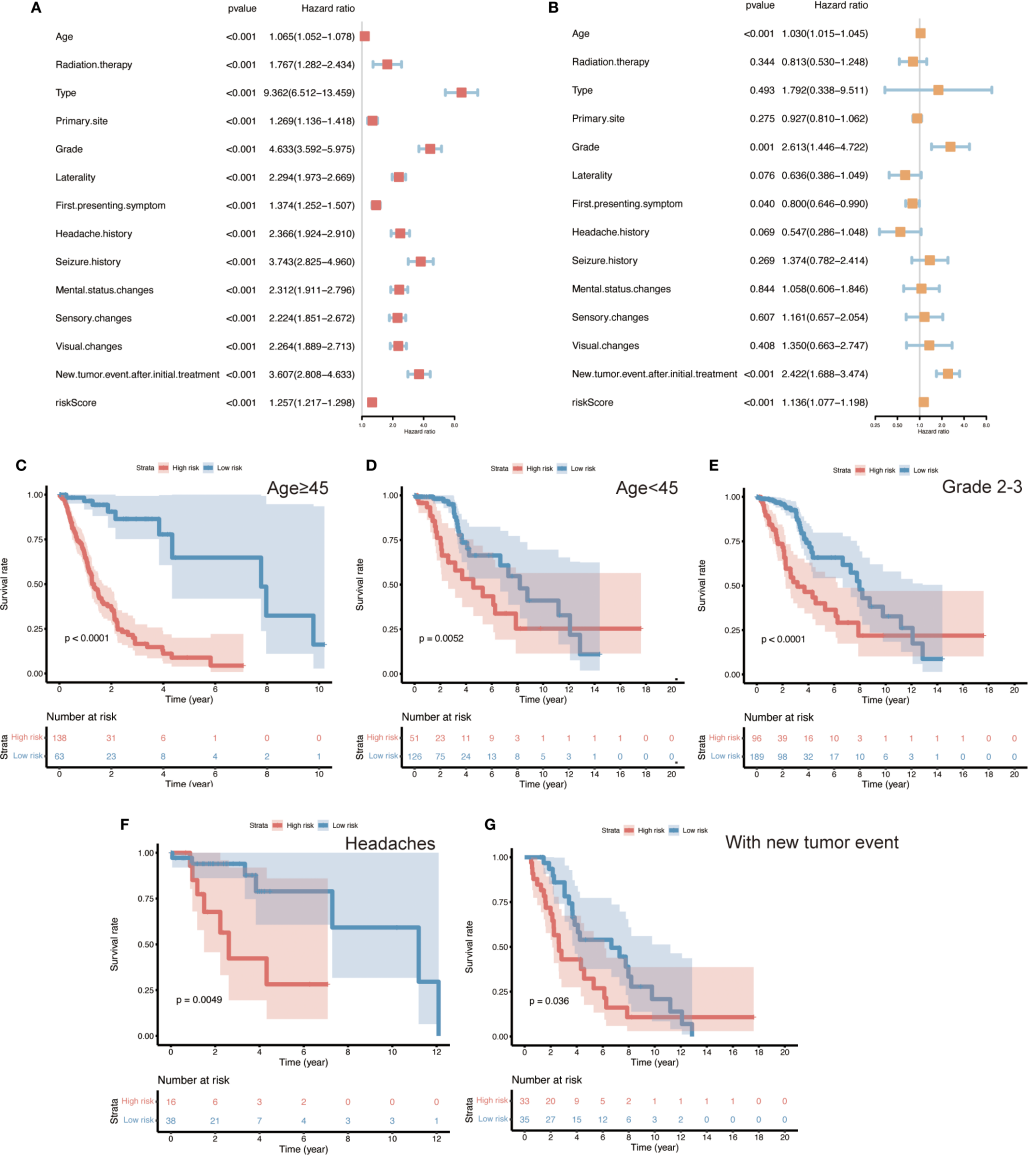
Identification of independent prognostic factors and risk subgroup survival analyses in glioma patients. **(A, B)** Univariate and multivariate Cox regression analyses of various clinicopathological variables (age, tumor grade, initial symptoms, new tumor events, and ten-gene risk score), identifying age, WHO grade, first symptom, occurrence of a new tumor event, and the risk score as independent prognostic factors (HRs with 95% CIs shown). **(C–G)** Kaplan–Meier overall survival curves for patient subgroups stratified by these independent factors, illustrating the prognostic impact of the ten-gene risk signature within each subgroup. Panels show stratifications by: age ≥45 years vs. <45 years, WHO Grade II–III tumors, headache vs. other initial symptoms, and presence vs. absence of a new tumor event. In all subgroups, high-risk patients have significantly shorter overall survival than low-risk patients. (Log-rank test, p < 0.05 for all comparisons).

Subsequently, univariate and multivariate Cox regression analyses were performed to assess the impact of clinicopathologic factors on the observed outcomes. The results identified age, grade, first symptom, neoplastic event, and risk score as independent risk factors (**Figures 5A, B**). Subsequent unfolding analyses for each subgroup identified age ≥45 years, WHO grade II - III, and headache as the first symptom with neoplastic event as independent risk factors (**Supplementary Figure 2**). The results of the internal and independent validation (**Supplementary Figure 3A–D**) further support the importance of age, grading, new tumor events, and risk scores as independent risk factors, particularly in high-risk populations.

### 3.7 Survival analysis in clinical subgroups

To further evaluate the performance of the risk model across different clinical contexts, we conducted survival analyses within various patient subgroups defined by the independent prognostic factors. In each subgroup, high-risk patients had significantly shorter overall survival than low-risk patients (**Figures 5C–G**; log-rank p < 0.05 for all comparisons). This pattern was observed among both younger patients (< 45 years) and older patients (≥ 45 years), in lower-grade gliomas (WHO II–III), in patients whose initial presenting symptom was headache, and in patients with a history of a new tumor event. These findings illustrate that the 10-gene risk signature retains prognostic relevance across a broad range of clinical subpopulations. It should be noted that the high-risk group consisted predominantly of WHO grade IV (GBM) cases – a subset of patients with uniformly poor outcomes. Consequently, a separate survival analysis within the GBM-only subgroup was deemed unnecessary, as nearly all GBM patients fell into the high-risk category; further stratification of this uniformly high-risk population would not be informative.

### 3.8 Functional enrichment analysis

**Figure 6:**
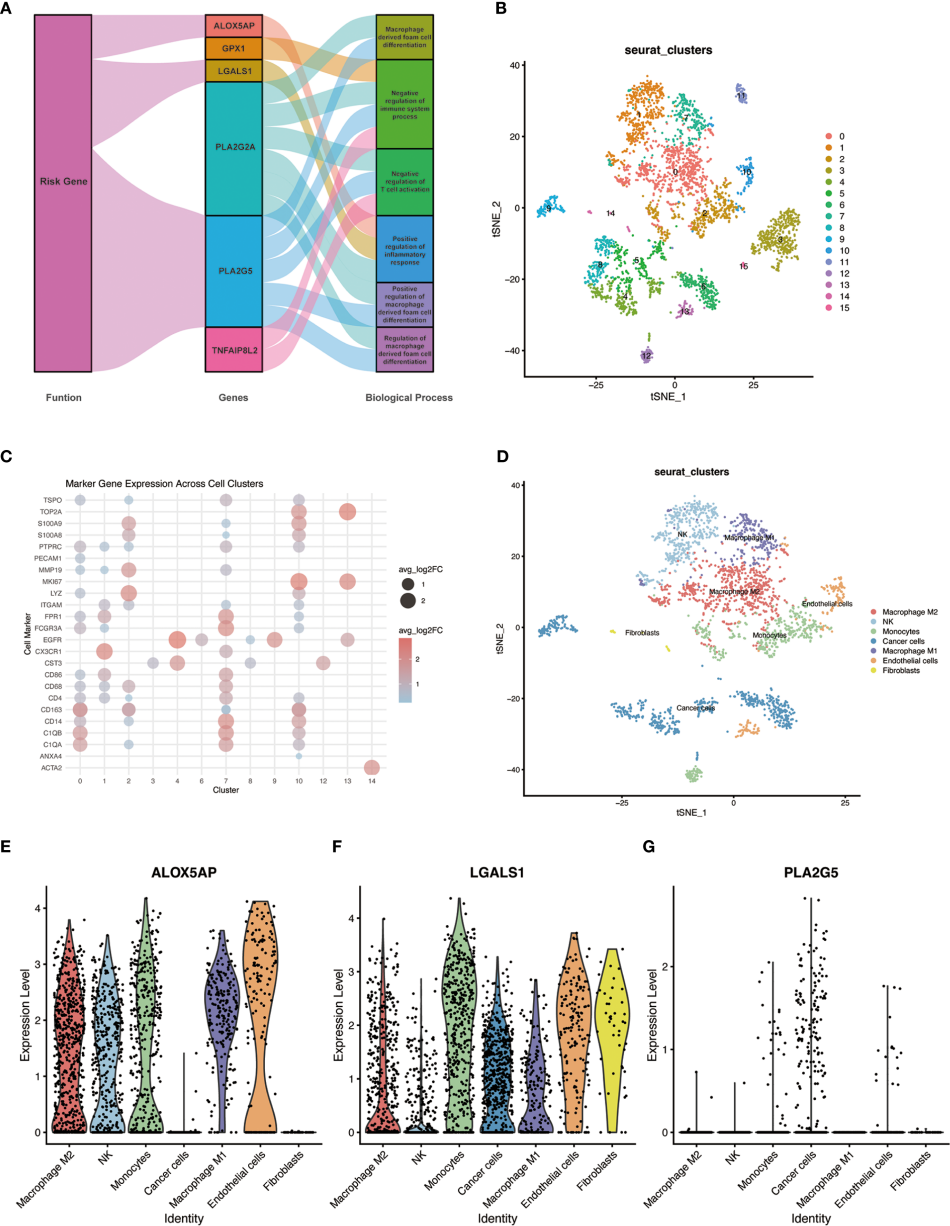
GO functional enrichment of risk genes and single-cell RNA sequencing analysis in GBM. **(A)** Network plot of enriched GO biological processes associated with the ten risk genes. Genes and GO terms are connected by lines indicating functional associations; line color and width reflect the relative magnitude and direction of each gene’s contribution to a given biological process. **(B)** UMAP plot of cells from primary GBM (single-cell RNA-seq dataset GSE84465), showing clustering of cells into distinct groups. **(C)** Dot plot of canonical cell-type marker gene expression across the identified cell clusters, used to determine the cell identity of each cluster. **(D)** UMAP plot of the GBM cells with clusters annotated by cell type (e.g., malignant cells, and various immune cell subtypes including distinct macrophage populations). **(E–G)** Violin plots showing the expression of three representative risk genes (*ALOX5AP*, *LGALS1*, and *PLA2G5*) across the annotated single-cell clusters; violin width represents the proportion of cells in the cluster expressing the gene, and color intensity denotes the average expression level. Notably, *ALOX5AP* is expressed in all major macrophage subclusters, *LGALS1* is predominantly expressed in M2-like macrophages, and *PLA2G5* shows low expression across all cell types.

To gain insight into the biological roles of the identified genes, we performed gene set enrichment analysis (GSEA) focusing on GO biological processes and relevant KEGG pathways (**Figure 6A**). This analysis revealed that the immune- and lipid metabolism-related genes are involved in key pathways related to immune regulation and macrophage function. In particular, pathways associated with macrophage-derived foam cell differentiation and immune response regulation were enriched in the high-risk group. Several of the 10 signature genes contributed to these enriched functions; for example, the risk genes *ALOX5AP*, *LGALS1*, and *PLA2G5* were highly expressed in high-risk tumors and were implicated in the GO terms identified. In addition, a few genes outside of the signature, such as PLA2G2A and TNFAIP8L2, were also found to play potential roles in these processes according to the enrichment results.

More specifically, the positive regulation of foam cell differentiation by PLA2G2A and PLA2G5 emerged as a notable function, which may shed light on underlying pathological mechanisms (analogous to processes in atherosclerosis) that could be at play in glioma macrophages. Meanwhile, TNFAIP8L2 was linked to the negative regulation of immune system processes and the positive regulation of inflammatory responses. The involvement of TNFAIP8L2 in these opposing regulatory functions highlights its potential importance in modulating the tumor’s immune microenvironment. Collectively, these enrichment findings suggest that the immune- and lipid metabolism-related genes identified in our study may influence glioma progression by affecting immune response pathways and macrophage behavior (e.g., polarization and foam cell formation), thereby contributing to the tumor’s biology and patient outcomes.

### 3.9 Single-cell RNA sequencing analysis

To investigate the cellular context of our key genes in the TME, we analyzed single-cell RNA sequencing (scRNA-seq) data from primary human GBM samples (GSE84465). Unsupervised clustering of the scRNA-seq data using the Seurat package identified multiple distinct cell clusters within the GBM specimens (**Figure 6B**). By referencing known cell-type markers from the literature and the CellMarker database, we determined the identity of each cluster and annotated the clusters accordingly (**Figures 6C, D**). Notably, several clusters corresponded to tumor-associated macrophages, representing different activation states of macrophages in the tumor. We next examined the expression of three representative genes from our risk signature – *ALOX5AP*, *LGALS1*, and *PLA2G5* – across the single-cell clusters. *ALOX5AP* was expressed in all of the major macrophage subpopulations, indicating broad activity in tumor-infiltrating macrophages. *LGALS1* expression was enriched in the M2-like macrophage clusters (an immunosuppressive phenotype), consistent with its putative role in promoting tumor progression. In contrast, *PLA2G5* showed uniformly low expression across all cell clusters, suggesting that at baseline no particular cell type in the TME highly expresses this gene (**Figures 6E–G**). These single-cell findings provide further evidence that the identified key genes – especially *LGALS1* and *ALOX5AP* – play functionally relevant roles in the context of macrophage polarization and function within the GBM TME.

### 3.10 Experimental validation of risk gene expression

**Figure 7:**
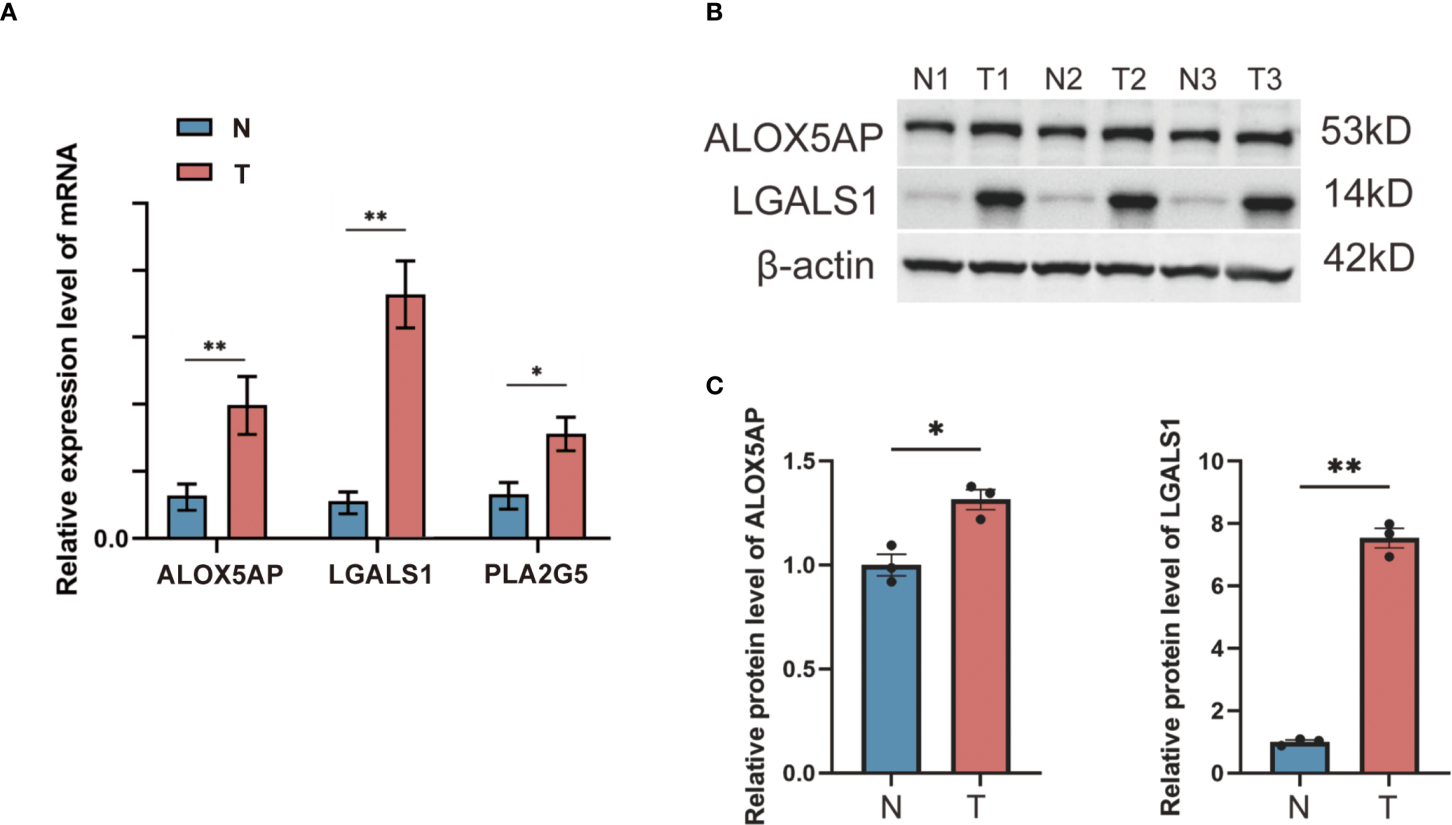
qRT-PCR and Western blot validation of risk gene expression in GBM vs. non-tumor brain tissues. **(A)** qRT-PCR measured the relative mRNA expression levels of three selected risk genes (*ALOX5AP*, *LGALS1*, and *PLA2G5*) in GBM tumor tissue compared to non-tumor brain tissue (β-actin as internal control; n = 15 per group). **(B)** Western blot detection of two representative risk gene products (ALOX5AP and LGALS1) in non-tumor (N) and GBM tumor (T) tissue samples, with β-actin as a loading control. **(C)** Densitometric quantification of the Western blot bands showing significantly elevated protein expression of ALOX5AP and LGALS1 in GBM tissues versus non-tumor tissues (*p < 0.05, **p < 0.01).

To validate the differential expression of key risk genes at the tissue level, we performed qRT-PCR and Western blot experiments using clinical samples. We examined GBM tumor tissues and non-tumor brain tissues for the expression of selected risk genes. The qRT-PCR results, based on 15 clinical GBM and paired non-tumor brain tissue samples, showed that the mRNA levels of *ALOX5AP*, *LGALS1*, and *PLA2G5* were significantly higher in GBM tumor tissues compared to non-tumor brain tissues (all *p* < 0.05) (**Figure 7A**). Consistently, Western blot analysis confirmed markedly up-regulated protein expression of ALOX5AP and LGALS1 in GBM tumors versus non-tumor tissues (**Figures 7B, C**). Densitometric quantification revealed a significant increase in ALOX5AP protein in GBM (p < 0.01) and in LGALS1 protein (p < 0.05) relative to normal brain samples. These findings corroborate that the identified risk genes are indeed overexpressed in GBM, supporting their proposed role in driving a high-risk phenotype.

### 3.11 Drug sensitivity analysis of *LGALS1*

**Figure 8:**
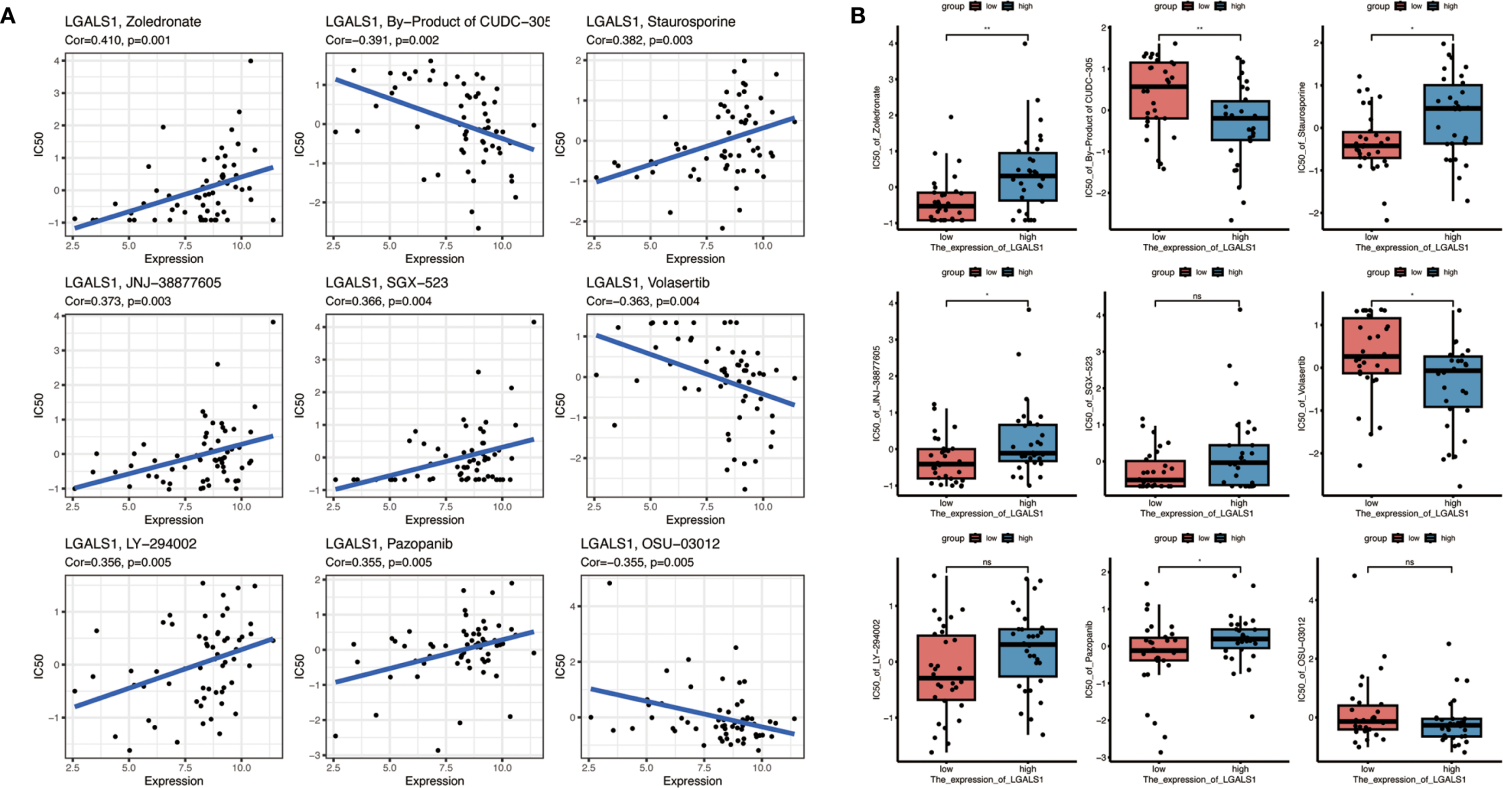
Drug sensitivity analysis related to LGALS1 expression. **(A)** Correlations between LGALS1 gene expression and the sensitivity of GBM cells to nine selected anticancer compounds (including FDA-approved drugs or those in clinical trials targeting the galectin-1 pathway). Higher *LGALS1* expression is significantly associated with increased sensitivity to four agents (e.g., zoledronate, staurosporine, JNJ-38877605, pazopanib) and with decreased sensitivity to two agents (a CUDC-305 metabolite and volasertib) (all correlation p < 0.05). **(B)** Comparison of drug sensitivity in the high-risk vs. low-risk patient groups for the same set of compounds. High-risk (*LGALS1*-high) patients tended to be more responsive to zoledronate, staurosporine, JNJ-38877605 and pazopanib, and less responsive to the CUDC-305 metabolite and volasertib, than low-risk patients, although these differences did not reach statistical significance (*p < 0.05, **p < 0.01, “NS” denotes not significant).

Given the elevated expression of *LGALS1* in M2-polarized macrophages and its association with poorer prognosis, we explored whether targeting *LGALS1* could have therapeutic implications. We screened 19 candidate compounds known to interact with galectin-1 (the protein encoded by *LGALS1*), with an emphasis on drugs that are either FDA-approved or in clinical trials (**Figure 8A**). From this screen, nine drugs were selected for detailed analysis of their sensitivity profiles in relation to *LGALS1* expression. Among these, *LGALS1* expression levels showed a significant positive correlation with tumor cell sensitivity to four agents – zoledronate, staurosporine, JNJ-38877605, and pazopanib – indicating that higher *LGALS1* might render tumors more susceptible to these drugs. Conversely, *LGALS1* expression was significantly negatively correlated with the sensitivity to two agents (a metabolite of CUDC-305 and the PLK inhibitor volasertib), suggesting that *LGALS1*-high tumors are relatively resistant to those treatments (**Figure 8A**). All of the above correlations were statistically significant (*p* < 0.05). In contrast, for three other tested drugs (SGX-523, LY-294002, and OSU-03012), no significant relationship between *LGALS1* expression and drug response was observed.

We further investigated drug sensitivities in the context of the risk stratification by comparing responses between high-risk and low-risk groups of patients (as defined by the 10-gene signature) (**Figure 8B**). The high-risk group, which exhibits higher *LGALS1* expression on average, showed trends consistent with the correlation analysis: for example, high-risk patients tended to be more responsive to zoledronate, staurosporine, JNJ-38877605, and pazopanib, whereas they appeared less sensitive to the CUDC-305 by-product and volasertib, compared to low-risk patients. However, these differences did not reach statistical significance in our cohort. Overall, this drug sensitivity analysis points to *LGALS1* as a promising therapeutic target. The identified *LGALS1*-associated compounds (such as zoledronate and pazopanib) may provide a basis for tailored treatment strategies in GBM, particularly for patients with tumors characterized by an immunosuppressive, M2-macrophage-rich microenvironment. These findings underscore the potential clinical utility of integrating our risk gene signature with drug response data to inform personalized therapy for GBM.

## 4 Discussion

Our study identified a set of immune- and lipid metabolism-related genes that are significantly associated with macrophage polarization and prognosis in GBM patients. By performing differential expression analysis, we uncovered 243 down-regulated and 443 up-regulated genes between immune and non-immune groups, with several of these genes playing a pivotal role in macrophage function and polarization. Among the 26 overlapping genes between immune-related and lipid metabolism-related genes, we identified 10 risk genes (*PLA2G5*, *LGALS1*, *GOS2*, *FABP5*, *PLBD1*, *ALOX5AP*, *ETNPPL*, *ACSL6*, *TSPOAP1*, and *TBXAS1*), which were associated with distinct prognostic outcomes.

The involvement of macrophages, particularly the polarization between M1 and M2 phenotypes, has profound implications for tumor progression (18, 19). M1 macrophages are typically associated with anti-tumor activities, producing pro-inflammatory cytokines like TNF-α and IL-12, and promoting tumor cell killing. In contrast, M2 macrophages, which are abundant in the TME of GBM, facilitate tumor growth by secreting anti-inflammatory cytokines (e.g., IL-10 and TGF-β), promoting angiogenesis, and remodeling the extracellular matrix (20–22). Our findings indicated that several risk genes, such as *LGALS1* and *PLA2G5*, were highly expressed in M2 macrophages, underscoring their potential role in promoting the immunosuppressive microenvironment that supports GBM progression. *LGALS1*, for example, has been shown to promote M2 polarization and contribute to immune evasion in GBM by inhibiting T-cell function and promoting regulatory T-cell activity (23, 24). Similarly, *ALOX5AP*, involved in leukotriene biosynthesis, has been linked to pro-tumorigenic functions in M2 macrophages (25, 26).

Lipid metabolism also plays a crucial role in regulating macrophage polarization and function in the TME. Lipid uptake, storage, and metabolism are significantly altered in TAMs, especially in M2-like macrophages, which utilize fatty acid oxidation (FAO) to sustain their immunosuppressive activities (27–29). Our study identified genes like *FABP5* and ACSL6, which are key players in lipid metabolism, as potential regulators of macrophage polarization in GBM. *FABP5*, a fatty acid-binding protein, has been implicated in the uptake and transport of fatty acids, particularly in M2 macrophages, where it supports FAO and oxidative phosphorylation (30). The upregulation of *FABP5* in high-risk GBM patients, as seen in our analysis, suggests that it may facilitate the metabolic reprogramming of TAMs to sustain tumor-promoting functions. Targeting these metabolic pathways may represent a promising strategy to modulate macrophage polarization and inhibit GBM progression.

In addition to macrophage polarization, our findings also shed light on the broader prognostic implications of immune and lipid metabolism-related genes in GBM. Survival analysis revealed that patients with higher expression levels of risk genes, such as *LGALS1* and *PLA2G5*, had significantly poorer outcomes, suggesting that these genes could serve as biomarkers for GBM prognosis. Furthermore, pathway enrichment analysis highlighted the involvement of these genes in immune regulation, inflammation, and lipid metabolism, reinforcing their role in shaping the TME and influencing tumor progression. The GO and KEGG pathway enrichment analysis revealed critical biological processes, including macrophage differentiation, lipid biosynthesis, and inflammatory responses, which are known to contribute to cancer progression (31, 32).

The therapeutic implications of our findings are significant. Given the crucial role of M2-like macrophages in supporting GBM, strategies aimed at reprogramming macrophages from the M2 to M1 phenotype have garnered increasing interest as potential therapeutic approaches (33, 34). Moreover, targeting lipid metabolism in TAMs represents another promising avenue for disrupting the tumor-promoting functions of these immune cells (35, 36). Drugs that inhibit fatty acid oxidation or interfere with lipid signaling pathways, such as inhibitors of *ALOX5AP* or *FABP5*, could serve as potential therapies for GBM by impairing the metabolic fitness of TAMs and reactivating anti-tumor immunity (37). Additionally, the identification of *LGALS1* as a potential therapeutic target opens up new possibilities for pharmacological intervention. Our drug sensitivity analysis revealed that *LGALS1* expression was correlated with sensitivity to several FDA-approved drugs, including zoledronate and staurosporine, highlighting the potential for repurposing existing drugs to target this immune regulatory protein in GBM.

This study has several limitations that should be acknowledged. First, we did not perform *in vivo* animal experiments to validate the therapeutic potential of the identified immune- and lipid metabolism–related genes. Such experiments will be important in the future to clarify their roles in macrophage polarization and tumor progression. Second, functional mechanistic studies such as gene knockdown, overexpression, flow cytometry, or cytokine profiling were not included; therefore, our conclusions remain correlative rather than causative. Future work incorporating these approaches will be valuable to provide direct causal evidence. Third, the number of clinical samples used for experimental validation was relatively limited, which may restrict the generalizability of the findings. Fourth, the single-cell RNA-seq analysis was based on four GBM specimens, which might not fully capture the extensive heterogeneity of the tumor microenvironment. Larger single-cell datasets could help to address this limitation in subsequent studies. Finally, the study relied heavily on public datasets (TCGA, CGGA, GEO), where sample collection and processing were beyond our control, potentially introducing bias. These limitations highlight the importance of further validation and the need for future studies to extend our findings.

Beyond glioblastoma, emerging evidence suggests that immune–metabolic interactions may also play important roles in other cancers. For example, in breast cancer and melanoma, lipid metabolic pathways have been shown to influence macrophage polarization and impact patient prognosis. This indicates that the immune–lipid axis identified in GBM may represent a broader mechanism relevant across multiple tumor types. Future studies comparing the prognostic and functional roles of these genes across different cancers will help to clarify their generalizability and potential as therapeutic targets.

Taken together, our findings highlight the critical role of immune- and lipid metabolism–related genes in shaping the glioblastoma microenvironment. These genes may substantially influence patient outcomes. By integrating bulk and single-cell transcriptomic analyses with experimental validation, we established a prognostic gene signature and provided preliminary evidence for its potential therapeutic relevance. Importantly, these results suggest that targeting immunometabolic pathways may offer new strategies for modulating macrophage polarization and improving GBM treatment. Looking forward, further mechanistic studies, *in vivo* validation, and larger independent cohorts will be essential to confirm the causal roles of these genes and to explore their translational potential. Ultimately, translating these insights into clinically actionable biomarkers and therapeutic strategies could contribute to more personalized and effective care for patients with glioblastoma.

## 5 Conclusions

This study highlights the prognostic significance of immune- and lipid metabolism-related genes in GBM, particularly in relation to macrophage polarization and tumor progression. The identification of key risk genes, such as *LGALS1* and *PLA2G5*, suggests novel therapeutic targets within immune modulation and lipid metabolism pathways. These findings provide a foundation for future research aimed at developing macrophage-targeted therapies and improving clinical outcomes for GBM patients.

## Data Availability

The datasets analyzed in this study are publicly available from the following repositories: The Cancer Genome Atlas (TCGA): https://portal.gdc.cancer.gov/. Chinese Glioma Genome Atlas (CGGA): http://www.cgga.org.cn/. Gene Expression Omnibus (GEO, GSE84465): https://www.ncbi.nlm.nih.gov/geo/query/acc.cgi?acc=GSE84465. The R scripts used for modeling and Seurat clustering are provided as **Supplementary Material**.
