# Supplementary material for: The role of immune- and lipid metabolism-related genes in macrophage polarization and prognosis of glioblastoma
Source: Front Oncol. 2025 Oct 14;15:1660754. doi: 10.3389/fonc.2025.1660754 (PMC12558797; doi:10.3389/fonc.2025.1660754)
Supplement: Supplementary file 1 [file DataSheet1.zip › Supplementary Materials/Supplementary Table 4_Primer sequences for PCR.docx]

**Table S4 Primer sequences for qRT-PCR**

| **Gene** | **Forward Primer (5'-3')** | **Reverse Primer (5'-3')** |
| --- | --- | --- |
| ALOX5AP | GCTCCCTGGCATACACAGAA | CCAGCAACGGACATGAGGAA |
| LGALS1 | ATGGCTTGTGGTCTGGTCG | CAGTCAAAGGCCACACATTTGA |
| PLA2G5 | ATGAAAGGCCTCCTCCCACT | AGGAGCAGAGGATGTTGGGA |

Complementary DNA (cDNA) was synthesized using the PrimeScript RT Reagent Kit (Takara, Japan; Cat# RR037A).
