# Supplementary material for: The role of immune- and lipid metabolism-related genes in macrophage polarization and prognosis of glioblastoma
Source: Front Oncol. 2025 Oct 14;15:1660754. doi: 10.3389/fonc.2025.1660754 (PMC12558797; doi:10.3389/fonc.2025.1660754)
Supplement: Supplementary file 1 [file DataSheet1.zip › Supplementary Materials/Supplementary Table 5_Primary antibodies for Western blot.docx]

**Table S5 Primary antibodies for Western blot**

| **Target** | **Antibody type** | **Supplier** | **Catalog number** | **Notes** |
| --- | --- | --- | --- | --- |
| ALOX5AP | Rabbit polyclonal | Sigma-Aldrich | HPA026592 | Primary antibody |
| LGALS1 | Mouse monoclonal (Clone 1E8 1B2) | Thermo Fisher | MA5-32779 | Primary antibody |
| β-actin | Mouse monoclonal | Proteintech | e66009-1-Ig | Loading control |
