# Supplementary material for: The role of immune- and lipid metabolism-related genes in macrophage polarization and prognosis of glioblastoma
Source: Front Oncol. 2025 Oct 14;15:1660754. doi: 10.3389/fonc.2025.1660754 (PMC12558797; doi:10.3389/fonc.2025.1660754)
Supplement: Supplementary file 1 [file DataSheet1.zip › Supplementary Materials/Supplementary_Figures.docx]

Supplementary Material

# Supplementary Figures

**
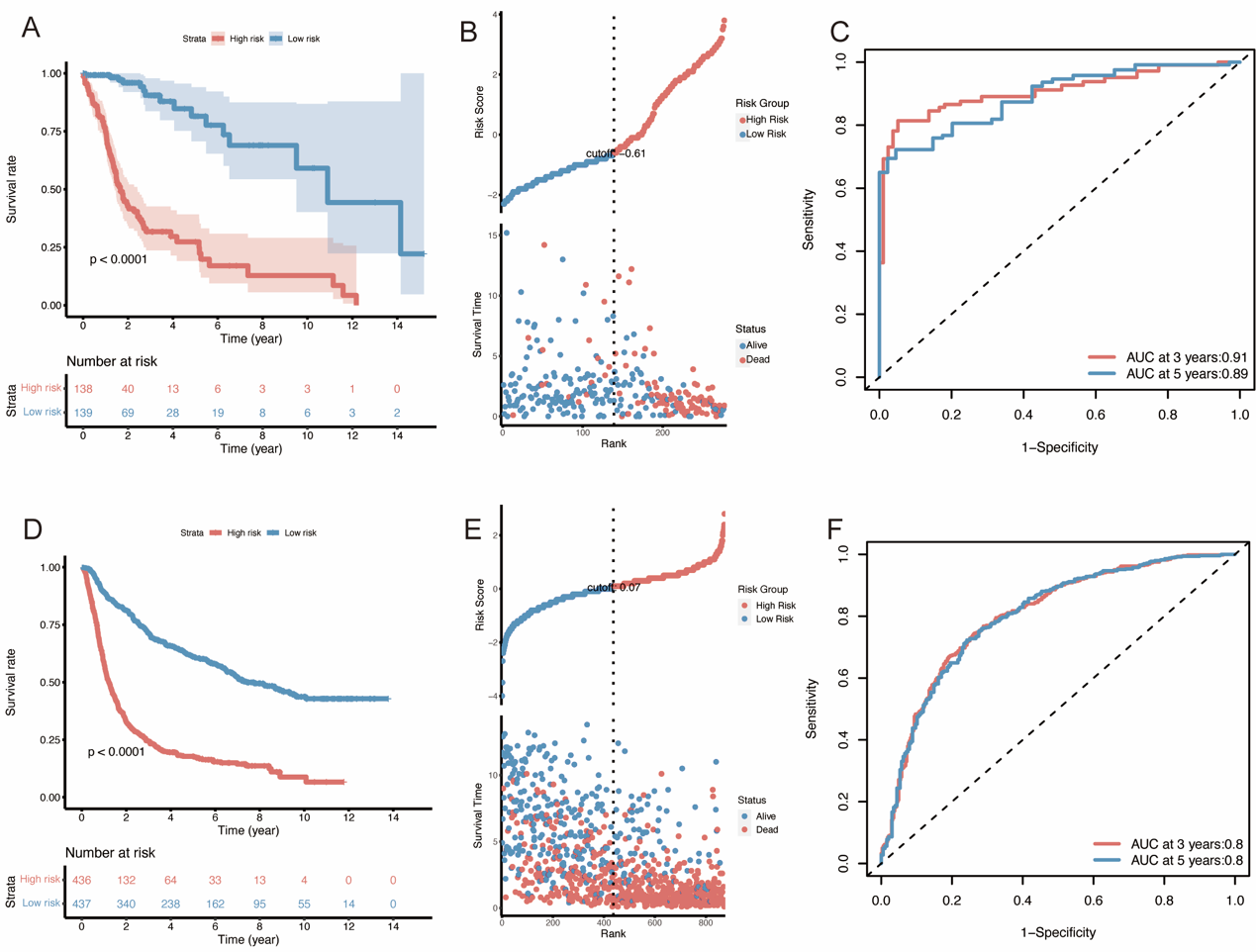
**

**Supplementary Figure 1.** Internal and external validation of the ten-gene prognostic signature. Kaplan–Meier survival curves are shown for the TCGA internal testing set and the CGGA external validation cohort, confirming that in both cohorts the high-risk group had significantly worse overall survival than the low-risk group (log-rank p < 0.01), consistent with the training set results.

**
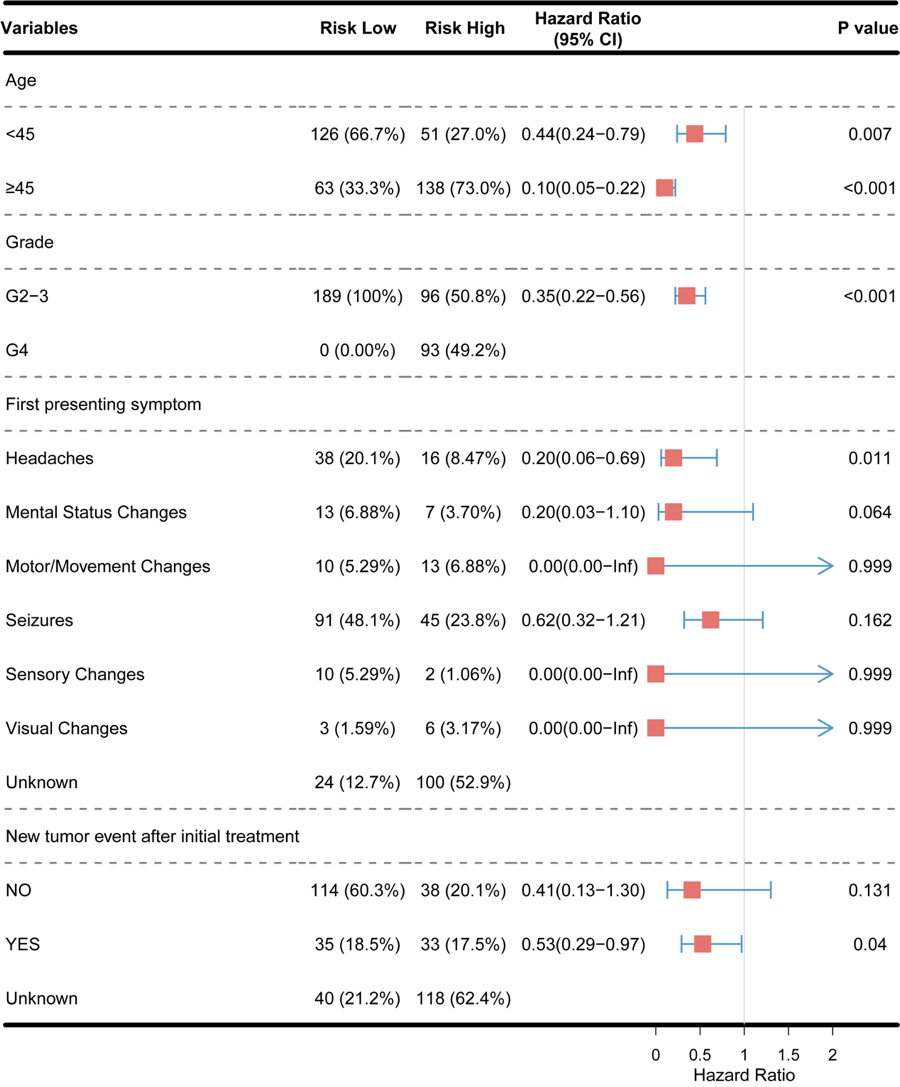
**

**Supplementary Figure 2.** Prognostic performance of the ten-gene risk signature in various patient subgroups. Kaplan–Meier survival curves illustrate the risk signature’s stratification within (A) patients aged <45 years, (B) patients aged ≥45 years, (C) patients with WHO grade II–III tumors, (D) patients whose initial symptom was headache, and (E) patients with a new tumor event. In every subgroup, high-risk patients have significantly shorter overall survival than low-risk patients (log-rank p < 0.05).

**
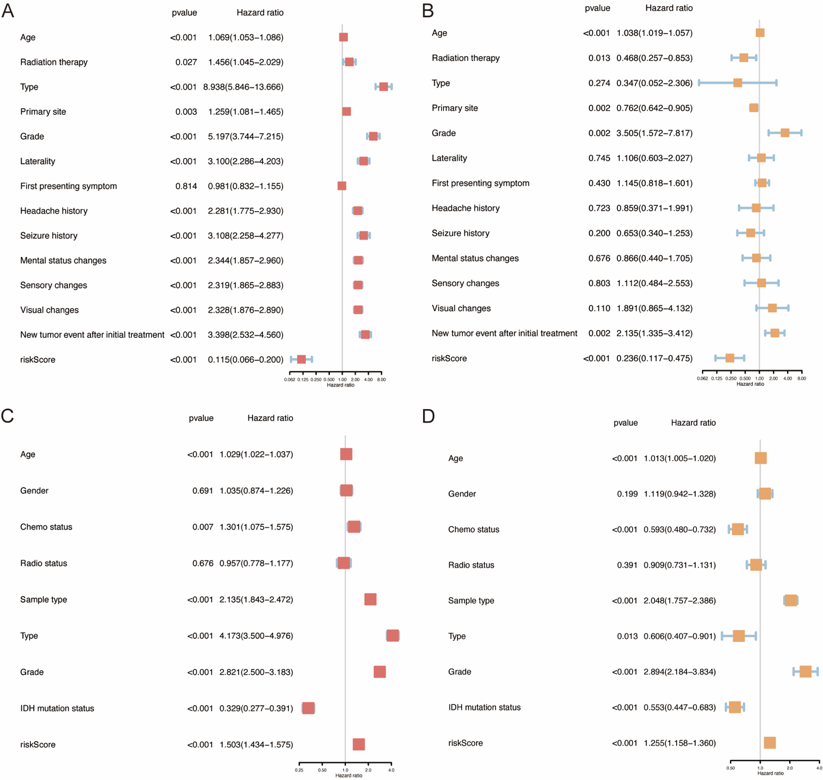
**

**Supplementary Figure 3. Validation of independent prognostic factors in an external cohort.** Multivariate Cox regression analysis in the CGGA dataset demonstrates that age, tumor grade, occurrence of a new tumor event, and the ten-gene risk score remain significant independent prognostic factors for overall survival (p < 0.05), supporting the broad applicability of these risk factors.
